# Use of Sodium-Chloride Difference and Corrected Anion Gap as Surrogates of Stewart Variables in Critically Ill Patients

**DOI:** 10.1371/journal.pone.0056635

**Published:** 2013-02-13

**Authors:** Jihad Mallat, Stéphanie Barrailler, Malcolm Lemyze, Florent Pepy, Gaëlle Gasan, Laurent Tronchon, Didier Thevenin

**Affiliations:** Department of Intensive Care Unit, Centre Hospitalier du Dr. Schaffner, Lens, France; University of Louisville, United States of America

## Abstract

**Introduction:**

To investigate whether the difference between sodium and chloride ([Na^+^] – [Cl^−^]) and anion gap corrected for albumin and lactate (AG_corr_) could be used as apparent strong ion difference (SID_app_) and strong ion gap (SIG) surrogates (respectively) in critically ill patients.

**Methods:**

A total of 341 patients were prospectively observed; 161 were allocated to the modeling group, and 180 to the validation group. Simple regression analysis was used to construct a mathematical model between SID_app_ and [Na^+^] – [Cl^−^] and between SIG and AG_corr_ in the modeling group. Area under the receiver operating characteristic (ROC) curve was also measured. The mathematical models were tested in the validation group.

**Results:**

in the modeling group, SID_app_ and SIG were well predicted by [Na^+^] – [Cl^−^] and AG_corr_ (R^2^ = 0.973 and 0.96, respectively). Accuracy values of [Na^+^] – [Cl^−^] for the identification of SID_app_ acidosis (<42.7 mEq/L) and alkalosis (>47.5 mEq/L) were 0.992 (95% confidence interval [CI], 0.963–1) and 0.998 (95%CI, 0.972–1), respectively. The accuracy of AG_corr_ in revealing SIG acidosis (>8 mEq/L) was 0.974 (95%CI: 0.936–0.993). These results were validated by showing excellent correlations and good agreements between predicted and measured SID_app_ and between predicted and measured SIG in the validation group (R^2^ = 0.977; bias = 0±1.5 mEq/L and R^2^ = 0.96; bias = −0.2±1.8 mEq/L, respectively).

**Conclusions:**

SID_app_ and SIG can be substituted by [Na^+^] – [Cl^−^] and by AG_corr_ respectively in the diagnosis and management of acid-base disorders in critically ill patients.

## Introduction

Disorders of acid-base balance are among the most common abnormalities seen in critically ill patients [1]. They are generally related to clinical outcomes and disease severity, especially for metabolic acidosis [1], [2]. Acid-base disorders are currently assessed by three different methods: the physiological approach [3], [4], the base excess approach [5], [6], and the physicochemical approach [7], [8]. The first two approaches, which are based on the analysis of plasma concentration of bicarbonate and standard base excess (SBE), and further completed by the use of plasma anion gap (AG) are the most widely methods used to evaluate the metabolic component of acid-base disturbances [9]. One advantage of these methods is that are easy to understand and apply in common clinical situations [9]. However, the SBE is a calculated figure derived from PaCO_2_ and arterial pH, but reliance on its use alone to quantify metabolic disturbances has a number of pitfalls. First, it cannot identify whether an acidosis is due to increased tissue acids, hyperchloremia, or a combination of both. Second, its calculation assumes normal plasma protein, which may limit its accuracy in the critically ill patients [10], [11]. On the other hand, AG is grossly underestimated in the presence of hypoalbuminemia, which is a frequent occurrence in critically ill patients [12].

An alternative evaluation is the mathematical model based on physiochemical principles described by Stewart [8], and modified by Figge [11], [13]. This theory states that three independent variables determine pH in plasma by changing the degree of water dissociation into hydrogen and hydroxide ions. The three independent variables are PaCO_2_; strong ion difference (SID), which is the difference between fully dissociated plasma anions and cations; and plasma weak acids, namely, albumin and phosphate. This method allows the clinician to quantify individual components of acid-base abnormalities and provides insight into their pathogenesis [14]. Many studies showed that this approach, compared to the traditional approaches, is the best to identify acid-base disorders in the population of critically ill patients [15], [16]. Nevertheless, the Stewart's approach is a time-consuming method and unsuitable at the bedside. Previous studies [16], [17] have shown that strong ion gap (SIG) could be substituted by the anion gap corrected for albumin and lactate (AG_corr_). Furthermore, recently, Nagaoka et al [18] suggested that the difference between sodium and chloride concentrations ([Na^+^] – [Cl^−^]) could be used as a surrogate of SID. Nevertheless, in these studies there was no independent sample of patients to validate these findings. Furthermore, the temporal evolution of these surrogates was not tested. Therefore, the aim of our study was to investigate whether AG_corr_ and [Na^+^] – [Cl^−^] difference could be used as SIG and SID surrogates respectively in critically ill patients, and to validate these results in a different sample of patients.

## Methods

### Ethics Statement

This study was approved by the Institutional Ethics Committee (comité d'éthique du centre hospitalier du Dr. Shaffner de Lens). As the blood tests and data collected in this study were all standard clinical practice, the requirement for informed written consent was waived, and only oral consent was obtained. There were no measures taken to document to verbal consent procedure; nevertheless, the entire consent procedure was submitted to the ethics committee before they approved this study. If the patient or his/her next of kin refused consent, patient's data were not entered into analysis.

### Clinical and laboratory data

A total of 341 patients admitted to the intensive care unit (ICU) of a general hospital from February to December 2011 were enrolled in the study. All data were retrieved from a prospectively collected database.

Clinical and laboratory data were collected from all patients at admission, and laboratory data were recorded again 24 hours later only in the sample of patients who served as a cross-validation group. The following clinical data were recorded: age, sex, Simplified Acute Physiology Score (SAPS II), cause of ICU admission, length of stay, and outcome. All arterial samples were analyzed in the central laboratory of the institution (Cobas 6000; Roche Diagnostics, Meylan, France). Na^+^, K^+^, and Cl^−^ were measured using the ion-selective electrode technique. Magnesium and phosphate concentrations were determined by colorimetric techniques (chlorophosphonazo 3 and ammonium molybdate complex colorimetric techniques, Roche Diagnostics respectively). Albumin was measured by immunoturbidimetry technique (Roche Diagnostics). Arterial blood gas analysis was performed using the GEM® Premier™ 3000 (Instrumentation Laboratory Co, Paris, France) with a preheparinized 3 mL blood gas syringe (RAPIDLyte®, Siemens Healthcare Diagnostic Inc, USA). Ionized calcium and lactate concentrations were determined with the GEM® Premier 3000. To ensure accurate measurement, the blood gas analyzer was calibrated several times a day.

### Acid-base calculations

Bicarbonate and SBE were calculated using the Henderson-Hasselbach and Van Slyke equations, respectively [3], [19]. The AG was calculated as follows [20]:
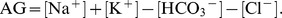
AG was corrected for the effect of abnormal albumin concentration [12] and lactate using the formula:
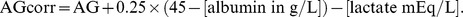



Physicochemical analysis was performed using the Stewart equations [8] modified by Figge et al. [11], [13] to consider the effects of plasma proteins. The apparent SID (SID_app_) is the difference between the sum of all measured strong cations and strong anions as follows (all concentrations in mEq/L):
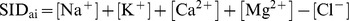
The strong ion gap (SIG) was calculated as follow:
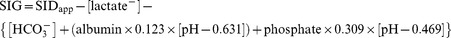
where HCO_3_^−^ and lactate are in mEq/L, albumin in g/L, and phosphate in mmol/L.

### Healthy volunteers and references data

Reference values for the SIG and SID_app_ were previously obtained from arterial blood samples of 13 healthy volunteers [16]. Data in the present study were similarly treated and analyzed. The normal values considered were those between the 2.5 and 97.5 percentiles of values from the healthy volunteers. Thus, the range of normal values for chloride was [99–104] mEq/L [16]. We defined SID_app_ acidosis and SID_app_ alkalosis, that are, metabolic acidosis and alkalosis attributable to disturbances in the inorganic ions, as a SID_app_ value below the 2.5th percentile (42.7 mEq/L) and as a SID_app_ value above 97.5^th^ percentile (47.5 mEq/L) respectively. Also, a SIG value above 8 mEq/L was considered elevated, indicating the presence of unmeasured anions [16].

### Patient subgroup division and sequence of analysis

Patients admitted during the first 5 months randomly constituted the modeling group (n = 161), and patients admitted during the last 6 months constituted the cross-validation group (n = 180). Data of the modeling group were used as the basis on which to build a mathematical linear model to derive an equation describing the relationship between SID_app_ and ([Na^+^] – [Cl^−^]) difference, and between SIG and AG_corr_.

The linear regression models were then tested in the cross-validation group. The regression coefficients produced by the analysis in the modeling group were applied to all initial measurements in a cross-validation group to calculate the predicted values of SID_app_ and SIG. The predicted values and actual values were then compared through correlation and agreement analysis. A large discrepancy between R^2^ (Pearson correlation coefficient) for the cross-validation and modeling groups indicated overfitting and lack of generalizability of the results of the analysis [21].

### Statistical analysis

The normality of data distribution was assessed using the Kolmogorov-Smirnov test. Proportions were used as descriptive statistics for categorical variables. Analysis of the discrete data was performed by Chi-square test. Continuous data that were not distributed normally were compared using the Mann-Whitney U test; otherwise the Student t test was applied. The mathematical model was built using simple linear regression analysis. Outliers were the cause of concern if more than 5% of cases have standardized residuals with an absolute value greater than 2 [22]. Influence cases were considered if Cook's distance values exceeding 1 [22]. The assumption of homoscedasticity was tested by plotted the standardized residuals values against the standardized predicted values of the dependent variable. To test the assumption of the normality of residuals, we looked at the histogram of the standardized residual. The assumption of independent residuals was tested with the Durbin-Watson test [22]. The correlation analysis was carried out with Pearson test. Agreement and limits of agreement of 95% were analyzed with the Bland-Altman plot [23] and intraclass correlation coefficient (ICC) [24] for continuous data, and with Kappa coefficient for categorical data [25]. Data were analyzed for the overall cross-validation group and three subgroups: a metabolic acidosis group (SBE<−2 mEq/L), a reference range group (−2 mEq/L≤SBE≤+2 mEq/L) and a metabolic alkalosis group (SBE>+2 mEq/L) [26]. Agreements were also analyzed in the septic shock group with or without acute kidney injury (serum creatinine>1.5 mg/dl) and with or without acute respiratory failure (pH<7.35 and PaCO_2_>45 mmHg). We used these groups to examine the possibility that different acid-base states may affect the agreement. Sensitivity and specificity, positive and negative likelihood ratios (LHR^+^ and LHR^−^, respectively), as well as accuracy (the area under the receiver operator characteristic (ROC) curve with a 95% confidence interval [CI]), were calculated to predict the accuracy of diagnosing SID_app_ acidosis, SID_app_ alkalosis, and increased SIG with each surrogate. We calculated the cutoff points with the greatest accuracy using Youden's index [27]. Statistical analyses were performed using SPSS (SPSS for windows release 17.0, Chicago, IL). A value of p<0.05 was considered statistically significant. All reported P values are 2-sided.

## Results

The general characteristics of patients, including reasons for ICU admission, clinical outcomes, ICU support measures, and laboratory data for the modeling and cross-validation groups are shown in Table 1.

**Table 1:** Patients' admission characteristics, support, outcomes, and laboratory data.

	Modeling group (n = 161)	Validation group (n = 180)	*p*
Age, y	64 [53–74]	62 [48–72]	0.3
SAPS II (mean ± SD)	56.5±19	54±17	1
Male sex, n (%)	89 (55)	106 (59)	0.5
Mechanical ventilation, n (%)	143 (89)	162 (90)	1
Renal replacement, n (%)	13 (8)	16 (9)	0.99
Vasopressors, n (%)	66 (41)	72 (40)	0.97
ICU survivors, n (%)	108 (67)	126 (70)	0.7
Length of ICU stays, day	11±10	10±9	0.96
Reason of admission, n (%)			
Respiratory failure	50 (31)	54 (30)	0.75
Septic shock	71 (44)	81 (45)	0.8
Postoperative	34 (21)	36 (20)	0.92
Others	6 (4)	9 (5)	0.99
Admission laboratory data			
Na^+^, mEq/L	138 [135–142] (124, 152)	138 [135–140] (106, 160)	0.43
K^+^, mEq/L	3.8 [3.4–4] (2.3, 5.3)	3.8 [3.6–4.3] (2.7, 7.5)	0.05
Cl^−^, mEq/L	105 [101–109] (84, 120)	103 [100–107] (69, 117)	0.014
Ca^2+^, mEq/L	2.26 [2.14–2.4] (1.34, 3.3)	2.22 [2.1–2.32] (1.34, 3.3)	0.019
Mg^2+^, mEq/L	1.72 [1.48–1.97] (0.9, 3.12)	1.64 [1.4–1.9] (0.9, 3.28)	0.019
PO_4_, mg/L	28 [21–38] (6, 91)	32 [24–43] (3, 104)	0.03
Albumin, g/L	24±5 (10, 37)	25±6 (10, 39)	0.12
Creatinine, mg/dL	1.15 [0.7–1.9] (0.3, 7.2)	1.2 [0.8–2.3] (0.25, 7.3)	0.3
Creatinine>2.5 mg/dL, n (%)	29 (18)	39 (22)	0.5
Lactate, mEq/L	1.2 [0.8–1.7] (0.3, 15)	1.3 [0.9–2.2] (0.2, 15	0.13
pH	7.41 [7.35–7.45] (7.03, 7.65)	7.39 [7.33–7.46] (6.92, 7.68)	0.037
SID_app_, mEq/L	40.6 [38–43.7] (29, 60)	42 [39–44.4] (27, 61)	0.02
SIG, mEq/L	6.5 [4–9] (−6, 21)	6.8 [4–10.6] (−8, 22)	0.11
PaCO_2_, mmHg	37 [32–45] (19, 100)	38 [33–44] (19, 123)	0.38
AG_corr_, mEq/L	16.2 [13.2–18.7] (4, 30)	16.8 [14–20.5] (2.5, 32)	0.03
[Na^+^] – [Cl^−^], mEq/L	33 [30–36] (20, 53)	34 [31–37] (21, 54)	0.025
SBE, mEq/L	−0.55 [−3.6–4.3] (−23, 29)	−0.83 [−5–4] (−23, 29)	0.23

SAPS, simplified Acute Physiology Score; SID_app_, apparent strong ion difference; SIG, strong ion gap; AG_corr_, anion gap corrected for albumin and lactate; SBE, standard base excess. Values are expressed as medians [interquartile range, 25–75] and (minimum, maximum) unless otherwise stated.

### [Na^+^] – [Cl^−^] difference as a surrogate of SID_app_ in the modeling group

The R^2^ of the regression analysis in the modeling group with SID_app_ as a dependent variable and [Na^+^] – [Cl^−^] difference as independent variable was 0.973 with the F statistics of 5722.33 (*P*<0.0001). There were 9 cases out of 161 (5.5%) with standardized residuals outside the limits of ±2. Therefore, our sample appears to conform to what we would expect for a fairly accurate model. Moreover, none of these 9 cases had a Cook's distance greater than 1 and so none of the cases had an undue influence on the model. The points of the graph of standardized residuals against standardized predictive values were randomly and evenly dispersed throughout the plot, demonstrating that the assumption of homoscedasticity had been met. Moreover, the histogram of the standardized residuals looked like a bell-shaped curve indicating that the assumption of linearity had also been met. The Durbin-Watson test of the model was 1.9 indicative of the independence of residuals. The equation of the model can be written as follows:
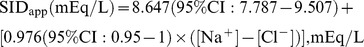
The effect of SID_app_ on SBE (R^2^ = 0.57) was not different from that of [Na^+^] – [Cl^−^] on SBE (R^2^ = 0.56).

The accuracy of [Na^+^] – [Cl^−^] to diagnose SID_app_ acidosis and alkalosis was excellent (0.992; 95%CI: 0.963–1 and 0.998; 95%CI: 0.972–1, respectively). Youden's index was used to determine that the best values of [Na^+^] – [Cl^−^] to predict SID_app_ acidosis (<42.7 mEq/L) and alkalosis (>47.5 mEq/L) were 34 mEq/L and 38 mEq/L, respectively. A value of [Na^+^] – [Cl^−^]≤34 mEq/L predicted SID_app_ acidosis (n = 110) with sensitivity of 94.5% (95% CI: 88.5–98), specificity of 98% (95% CI: 89–100), LHR^+^ of 52 (95% CI: 13.16–205.4) and LHR^−^ of 0.06 (95% CI: 0.026–0.12). A value of [Na^+^] – [Cl^−^]>38 mEq/L predicted SID_app_ alkalosis (n = 15) with sensitivity of 100% (95% CI: 77–100), specificity of 96 (95%CI: 86–96), LHR^+^ of 24.3 (95% CI: 2–277), and LHR^−^ of 0. Table 2 shows the accuracy, sensitivity, specificity, LHR^+^, and LHR^−^ of SID_app_ surrogate in a subgroup of patients with hyponatremia ([Na^+^]<135 mEq/L).

**Table 2:** Sensitivity, specificity, likelihood ratios, and accuracy of apparent strong ion difference (SID_app_) surrogate in the presence of Hyponatremia (Na^+^<135 mEq/L) (n = 57).

	SID_app_ acidosis n = 43 (75.4)	SID_app_ alkalosis n = 2 (3.5)
[Na^+^] – [Cl^−^], cutoff	≤34 mEq/L	>38 mEq/L
Sensitivity (%), (95% CI)	95 (84–99)	100 (19–100)
Specificity (%), (95% CI)	93 (66–99)	98 (90–100)
LHR^+^, (95% CI)	13.35	55
LHR^−^, (95% CI)	0.05	0
Accuracy, (95% CI)	0.986 (0.911–0.997)	1 (0.937–1)

LHR^+^, positive likelihood ratio; LHR^−^, negative likelihood ratio; CI, confidence interval.

### AG_corr_ as a surrogate of SIG in the modeling group

The R^2^ of the regression analysis in the modeling group with SIG as a dependent variable and AG_corr_ as independent variable was 0.96 with the F statistics of 3606 (*P*<0.0001). There were 10 cases out of 161 (6%) with standardized residuals outside the limits of ±2. Moreover, none of these 10 cases had a Cook's distance grater than 1. The Durbin-Watson test of the model was 2, and the pattern of the points in the standardized residuals against standardized predictive values plots was indicative that the assumptions of linearity and homoscedasticity had been met. The equation of the model can be written as follows:
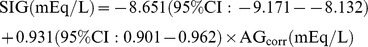
The effect of SIG on SBE (R^2^ = 0.61) was also not different from that of AG_corr_ on SBE (R^2^ = 0.58).

The accuracy of AG_corr_ to diagnose SIG acidosis was excellent (0.974; 95%CI: 0.936–0.993). The best value found using Youden's index for AG_corr_ to predict SIG acidosis (>8 mEq/L) was 17 mEq/L. A value of AG_corr_>17 mEq/L predicted SIG acidosis with sensitivity of 95% (95% CI: 86–99), specificity of 93% (95% CI: 86–97), LHR^+^ and LHR^−^ of 13.5 (95% CI: 6.5–27.2) and 0.05 (95% CI: 0.018–0.16); respectively.

### Validation of the SID_app_ and SIG surrogates in the cross-validation group

**Figure 1:**
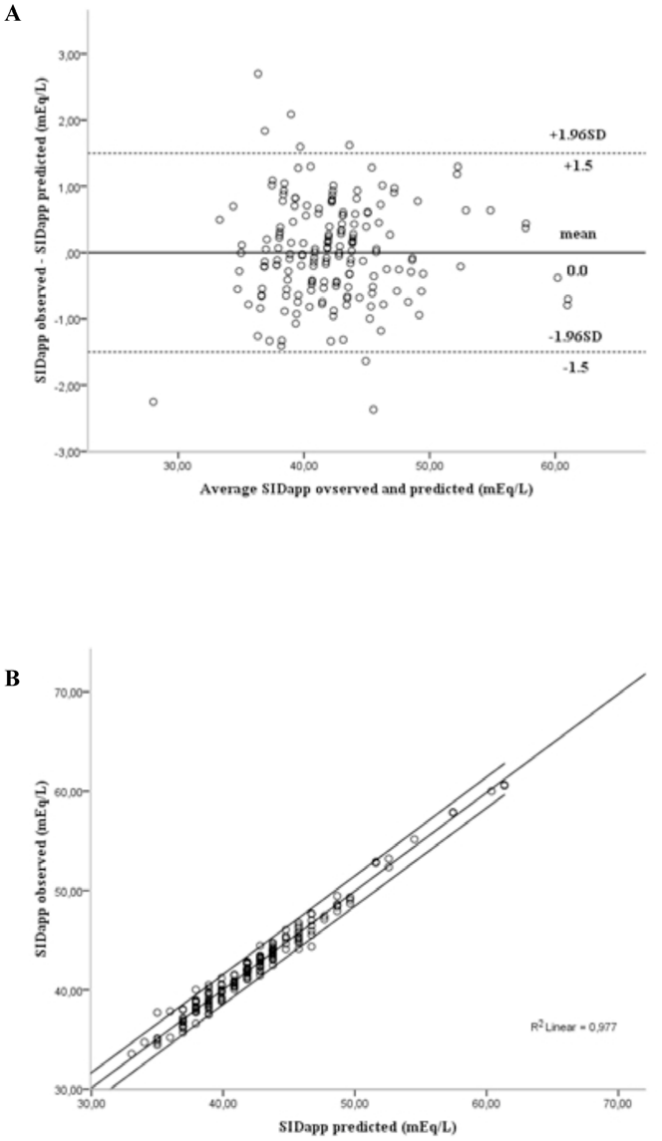
Correlation and agreement between observed and predicted apparent strong ion difference (SIDapp) in the cross-validation group. Panel A shows the agreement between observed and predicted SIDapp (bias = 0, limits of agreement 95% = −1.5 to 1.5 mEq/L). Panel B shows the correlation between observed and predicted SIDapp (R2 = 0.977, P<0.0001).

**Figure 2:**
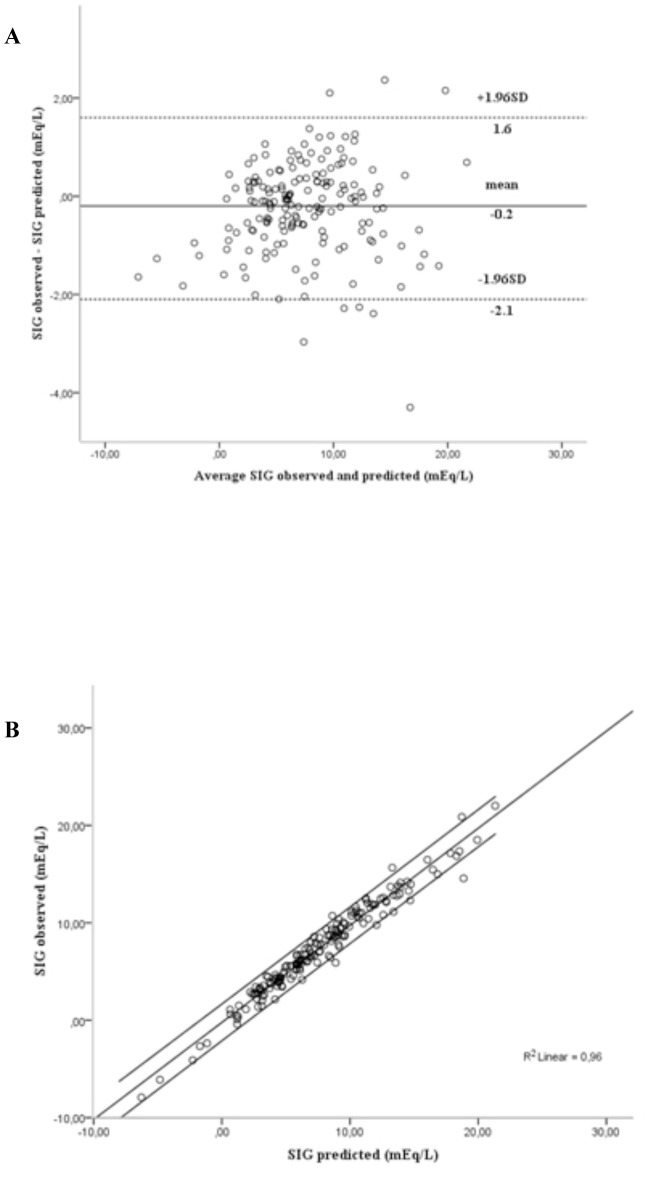
Correlation and agreement between observed and predicted strong ion gap (SIG) in the cross-validation group. Panel A shows the agreement between observed and predicted SIG (bias = −0.2, limits of agreement 95% = −2.1 to 1.6 mEq/L). Panel B shows the correlation between observed and predicted SIG (R2 = 0.96, P<0.0001).

Applying the linear regression models to the cross-validation group, a predictable measure of SID_app_, and a predictable measure of SIG were calculated. The similarities between predicted and actual values of SID_app_ and between predicted and actual values of SIG were demonstrated by the excellent correlations and agreements between them (Figure 1 and 2). Furthermore, the ICC was 0.988 (95% CI: 0.984–0.991; P<0.0001) between predicted and observed values of SID_app_, and 0.980 (95% CI: 0.973–0.985; P<0.0001) between predicted and observed values of SIG. These findings were similar in the three subgroups according to SBE (Table 3) and in septic shock patients with and without acute kidney injury and with and without acute respiratory failure (Table 4). By using all first and second data in the cross-validation group, we found an excellent correlation between changes in measured SIG and predicted SIG (R^2^ = 0.94; *P*<0.0001), and between changes in measured SID_app_ and predicted SID_app_ (R^2^ = 0.93; *P*<0.0001).

**Table 3:** Subgroups analysis of acid-base variables, agreements and intraclass correlation coefficients between observed and predicted values of SID_app_ and of SIG, and kappa coefficients between SID_app_ and its surrogate and between SIG and its surrogate in the cross-validation group.

	Metabolic acidosis (n = 75)	Reference range (n = 44)	Metabolic alkalosis (n = 61)
pH	7.32 (6.92–7.47)	7.4 (7.29–757)	7.45 (7.28–7.68)
HCO_3_, mEq/L	19 (2–25)	24 (21–28)	29 (25–56)
PaCO_2_, mmHg	34 (19–123)	39 (25–57)	42 (28–118)
[Na^+^] – [Cl^−^], mEq/L	31 (21–41)	34 (27–42)	36 (29–54)
SID_app_, mEq/L	39 (27–48)	42 (35–49)	44 (37–60)
SIG, mEq/L	11 (3–22)	6.5 (3–16.5)	4 (−8–14.6)
AG_corr_, mEq/L	21 (12–32)	16.4 (12–26.5)	14 (3.5–29.5)
Albumin, g/L	23 (10–39)	24 (17–38)	26 (14–39)
ICC between observed and predicted SID_app_, 95%CI	0.968 (0.950, 0.980)	0.984 (0.970, 0.991)	0.992 (0.986, 0.995)
Agreement between observed and predicted SID_app_	0.1 (−1.7, 1.9)	−0.12 (−1.18, 0.95)	−0.02 (−1.37, 1.33)
ICC between observed and predicted SIG, 95%CI	0.969 (0.951, 0.981)	0.955 (0.919, 0.976)	0.966 (0.943, 0.979)
Agreement between observed and predicted SIG	−0.1 (−2.1, 1.8)	−0.2 (−1.83, 1.43)	−0.4 (−2.3, 1.5)
Kappa between SID_app_ and [Na^+^] – [Cl^−^], 95%CI	0.848 (0.702, 0.993)	0.755 (0.553, 0.957)	0.819 (0.692, 0.945)
Kappa between SIG and AG_corr_, 95%CI	0.735 (0.533, 0.938)	0.807 (0.627, 0.987)	0.743 (0.469, 1)

SID_app_, apparent strong ion difference; SIG, strong ion gap; AG_corr_, anion gap corrected for albumin and lactate; ICC, intraclass correlation coefficient; CI, confidence interval. Metabolic acidosis = SBE<−2 mEq/L, reference range = −2 mEq/L≤SBE≤+2 mEq/L, and metabolic alkalosis = SBE>+2 mEq/L. Agreement is expressed as bias, (95% limits of agreement). All others data are expressed as median with range (minimum, maximum).

**Table 4:** Subgroups analysis in the septic shock patients of the cross-validation group according to the presence of acute kidney injury and of acute respiratory failure.

	AKI (n = 32)	Non-AKI (n = 49)	ARF (n = 20)	Non-ARF (n = 61)
ICC between observed and predicted SID_app_, 95%CI	0.990 (0.983, 0.993)	0.989 (0.983, 0.992)	0.996 (0.993, 0.998)	0.982 (0.974, 0.987)
Agreement between observed and predicted SID_app_	0.21 (−1.20, 1.62)	−0.13 (−1.62, 1.35)	0.27 (−0.80, 1.35)	−0.06 (−1.61, 1.61)
ICC between observed and predicted SIG, 95%CI	0.981 (0.967, 0.989)	0.981 (0.970, 0.988)	0.977 (0.956, 0.988)	0.976 (0.967, 0.983)
Agreement between observed and predicted SIG	−0.30 (−1.44, 0.84)	−0.21 (−1.73, 1.32)	−0.62 (−2.34, 1.10)	−0.15 (−1.99, 1,7)
Kappa between SID_app_ and [Na^+^] – [Cl^−^], 95%CI	0.879 (0.704, 1)	0.817 (0.670, 0.964)	0.918 (0.689, 1)	0.812 (0.679, 0.944)
Kappa between SIG and AG_corr_, 95%CI	0.732 (0.521, 0.931)	0.842 (0.775, 1)	0.817 (0.584, 1)	0.944 (0.752, 1)

AKI, acute kidney injury; ARF, acute respiratory failure, SID_app_, apparent strong ion difference; SIG, strong ion gap; AG_corr_, anion gap corrected for albumin and lactate; ICC, intraclass correlation coefficient; CI, confidence interval. Agreement is expressed as bias, (95% limits of agreement).

The best cutoff values of [Na^+^] – [Cl^−^] to diagnose SID_app_ acidosis and alkalosis found in the model group were applied to the cross-validation group for validation. In the first measurements of the cross-validation group, kappa coefficient between [Na^+^] – [Cl^−^] values [low (≤34 mEq/L), normal (35–38 mEq/L), high (>38 mEq/L)] and SID_app_ values [low (<42.7 mEq/L), normal (42.7–47.5 mEq/L), high (>47.5 mEq/L)] was very good (0.842, 95%CI: 0.786–0.916; *P*<0.0001). The same analysis was done between AG_corr_ (cutoff value>17 mEq/L) and SIG values (>8 mEq/L) in the first measurements of the cross-validation group. Kappa coefficient between these variables was also very good (0.85, 95%CI: 0.773–0.928; *P*<0.0001).

Negative values of SIG were found in 10 (3%) of the 341 patients. All these patients had severe metabolic alkalosis (SBE = 22.3±6 mEq/L and [HCO_3_^−^] = 48±7 mEq/L), associated with hypochloremia ([Cl^−^] = 93±7 mEq/L).

## Discussion

The main findings of this study were that: 1) SID_app_ and SIG were well predicted by [Na^+^] – [Cl^−^] difference and AG_corr_ values respectively. These were confirmed by the excellent correlation and good agreement found between measured and predicted values of Stewart's variables in the cross-validation group, and in the three subgroups classified according to SBE. 2) The accuracies of [Na^+^] – [Cl^−^] and AG_corr_ in revealing metabolic disturbance according to SID_app_ and to SIG (respectively) were very high. Furthermore, these findings were confirmed by finding that the kappa coefficients between [Na^+^] – [Cl^−^] and SID_app_ values, and between AG_corr_ and SIG values were very good in the cross-validation group.

Serum bicarbonate, SBE, and AG are commonly used to assess acid-base disorders [9]. However, it is recognized that this method can fail to identify the complex metabolic disturbances seen in critically ill patients, and so is generally inadequate in explaining them [14]. An alternative approach is the application of basic physicochemical principles of aqueous solutions to blood. Stewart method allows for the quantification of pH variations in proportion of changes in the independent variables [8]. However, Stewart developed his mathematical model in a flask, and there are certain points to note when applying this model to human plasma. First, the PaCO_2_ is an independent variable in an “open” system, where the total carbon dioxide is not fixed because it is in equilibrium with alveolar gas. However, this does not strictly apply to venous blood and fluid within the tissues, where the system is closed and the total carbon dioxide content rather than PaCO_2_ is the independent variable [9]. Second, no quantitative assessment of the secondary responses to primary changes in acid-base status is offered by the physicochemical approach [28]. Nevertheless, several studies [15], [16], [17], [29] have demonstrated that the Stewart's approach to acid-base disturbances allows the differentiation between tissue acidosis and hyperchloremic acidosis, and then results in identification of more patients with major acid-base disorders than the traditional evaluation.

Quantitatively, a change in the strong ion composition leading to lower SID will increase [H^+^] and causing SID_app_ acidosis while an increase in SID will decrease [H^+^] and causing SID_app_ alkalosis. Hyperchloremic acidosis therefore causes acidosis by decreasing SID_app_ and not through hyperchloremia alone. Indeed, normochloremia can occur alongside hyponatremia and result in acidosis by decreasing SID_app_, and hypernatremia can occur alongside hyperchloremia without acidosis (no change in SID_app_) [30]. At the other end of the spectrum, alkalosis may thus occur with both hypochloremia and hyperchloremia, with the latter occurring in the presence of greater hypernatremia (greater SID_app_) [31]. These highlight the importance of SID_app_ in our understanding and management of complex acid-base disorders in critically ill patients.

Nevertheless, ionized calcium and magnesium concentrations (2 components of SID_app_) are not included in routine chemistry profiles in ICU. Moreover, calculation of SID_app_ is time-consuming and is therefore not convenient for use in daily practice. Thus, a simplified equation is suitable for use at the bedside. Chloride and sodium are the most abundant extracellular ions and then the major contributors to the SID_app_. Previous studies have shown that in plasma, the SID_app_ is largely the difference between sodium cations and chloride anions [32], [33]. Recently, the difference between [Na^+^] and [Cl^−^] was found to have a good correlation and short limit of agreement with SID_app_ [18]. However, in these studies there was no independent sample of patients to validate these findings. In our study, we built a linear regression equation to assess the relationship between SID_app_ and [Na^+^] – [Cl^−^] in the modeling group of patients. We found that SID_app_ can well be predicted from [Na^+^] – [Cl^−^]. Furthermore, the effect of SID_app_ on SBE was not different from that of [Na^+^] – [Cl^−^] on SBE.

In the same way, we found that SIG can well be predicted from AG_corr_, and the effect of SIG on SBE was also not different from that of AG_corr_ on SBE. Our findings are in line with previous researches [16], [17], [26], which found a high correlation and good agreement between AG_corr_ and SIG.

To test the mathematical models resulting from the modeling group analysis, the equations that have been built were used to predict the values of SID_app_ and SIG in the cross-validation group. We found an excellent correlation and good agreement and precision between predicted and measured SID_app_ and between predicted and measured SIG (Figure 1 and 2). These were still true in the three subgroups classified according to SBE (Table 3). In addition, the temporal evolution of the predicted SID_app_ and SIG was well correlated with that of the observed SID_app_ and SIG. Therefore, by these findings we have demonstrated that these models are accurate and can be generalized to other samples.

Moreover, the accuracies of [Na^+^] – [Cl^−^] and AG_corr_ in revealing SID_app_ metabolic disorders and SIG acidosis (respectively) were high in the modeling group. Furthermore, we validated the cutoff values by finding a very good kappa coefficient between [Na^+^] – [Cl^−^] and SID_app_ values, and between AG_corr_ and SIG values in the cross-validation group. Our results are in accordance with those of Nagaoka et al. [18]. Nevertheless, the discrepancy between the cutoff values of [Na^+^] – [Cl^−^] to predict SID_app_ acidosis in that study (32.5 mEq/L) and in ours could be explained by the poor reproducibility of electrolyte measurements between different laboratory analyzers [34].

Patients with hyponatremia can have SID_app_ acidosis with normal serum chloride levels or can have hypochloremia without SID_app_ alkalosis [30]. The cutoff values of [Na^+^] – [Cl^−^] retrieved from the ROC curve were able to identify SID_app_ acidosis and alkalosis with high specificity and sensitivity in patients with hyponatremia (Table 2). We found only 10 patients with hypernatremia (Na^+^>145 mEq/L) that is why we could not do the same analysis with these patients.

The observed negative values of SIG in our patients may mean an error in measurement or the presence of unmeasured cations, which is rare even in critically ill patients. We think that laboratory error was unlikely since: (1) sodium and chloride were both measured using ion selective electrodes, and (2) no unexpected results were found in other patients. Moreover, all these patients suffered from severe metabolic alkalosis associated with hypochloremia. Therefore, the only feasible explanation for this rare finding was the accumulation of unmeasured cations. Medical literature involving unmeasured cations is poor [15], [35] and has been described in patients with chronic renal failure (accumulation of guanidines) [36], lithium intoxications [37], and paraproteinemias (positively charged gammaglobulins) [38]. In our patients, there was no history of medication abuse, and we did not investigate the presence of gammopathy.

Our study has several limitations. First, it has been done in a large sample of critically ill patients from a single unit. Our findings might not apply to other populations. However, our ICU admits a variety of medical and surgical patients, and our population is likely to be representative of other general ICU populations. Second, our results might not be applicable in patients with hypernatremia due to their small number.

## Conclusion

The present study demonstrates that SID_app_ and SIG can be substituted by the difference between [Na^+^] and [Cl^−^] and by the AG_corr_ respectively in the diagnosis and management of acid-base disorders in critically ill patients. In this manner, the use of these surrogates in metabolic acid-base disorders is fast and simple and may prevent the need of the complex calculations of Stewart's method.
